# Supplementary material for: Targeting Mitochondrial IDH2 Enhances Antitumor Activity of Cisplatin in Lung Cancer via ROS-Mediated Mechanism
Source: Biomedicines. 2023 Feb 7;11(2):475. doi: 10.3390/biomedicines11020475 (PMC9953680; doi:10.3390/biomedicines11020475)
Supplement: Supplementary file 1 [file biomedicines-11-00475-s001.zip › biomedicines-2143502-supplementary.pdf]

Figure S1

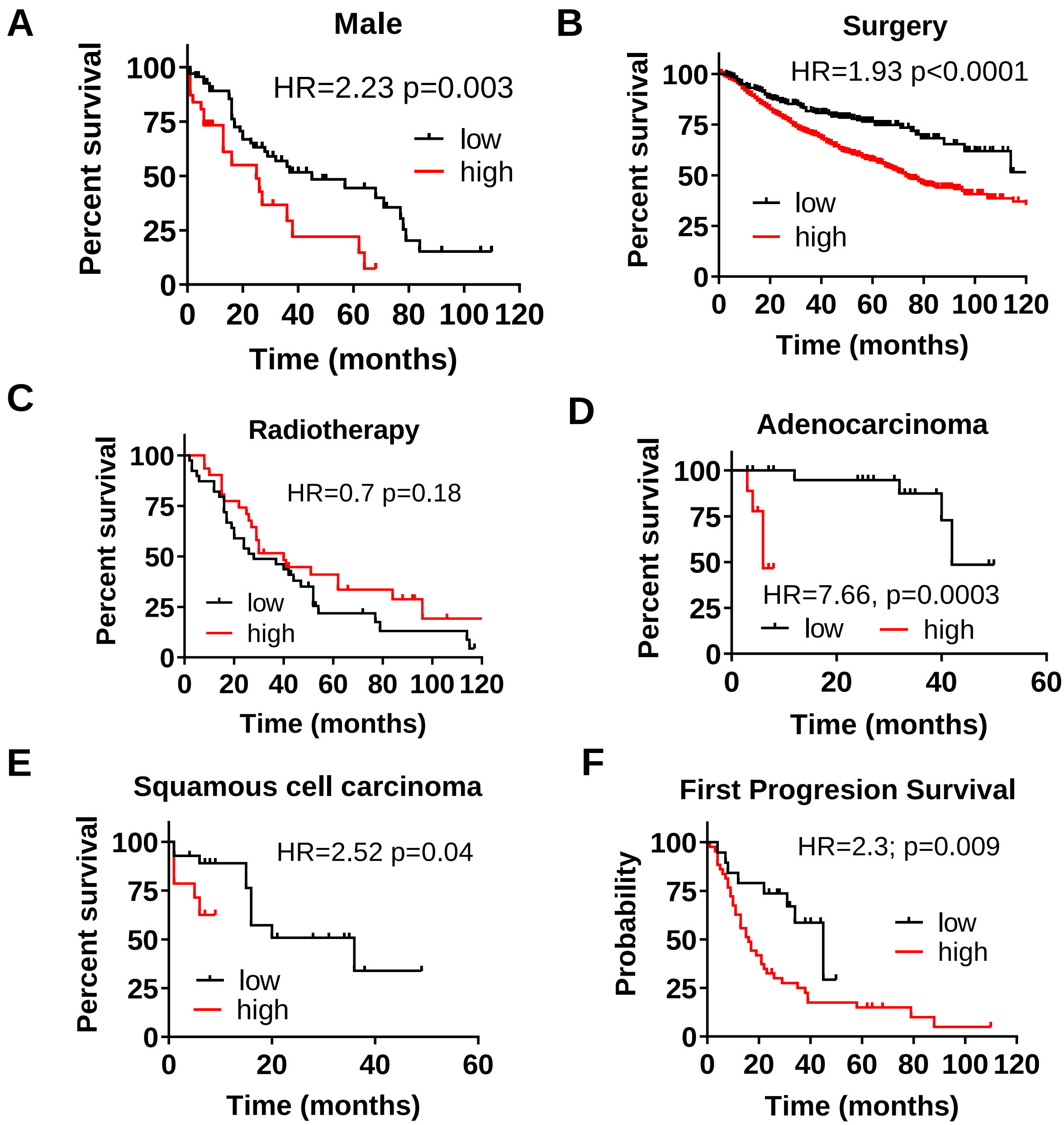

Figure S1. Relationship between IDH2 expression and lung cancer patient survival.(A) Kaplan-Meier analysis of overall survival of male lung cancer patients stratified by IDH2 expression levels. (B,C) Kaplan-Meier survival curves of lung cancer patients with surgery (B), or with radiotherapy treatment (C), stratified by IDH2 expression levels. The best performing threshold value of IDH2 expression was used as the cut-off (data was from Kmplot database). (D,E) Kaplan-Meier survival curves of lung cancer patient with lung adenocarcinoma (D), or squamous cell lung carcinoma patient (E), stratified by IDH2 expression. The best performing threshold value of IDH2 expression was used as the cut-off. (F) Kaplan-Meier analysis of first progression survival of male lung cancer patients, stratified by IDH2 expression. The best performing threshold value of IDH2 expression was used as the cut-off. All datasets were from Kmplot lung database (http://kmplot.com/analysis/index.php?p=service&cancer=lung).

Figure S2

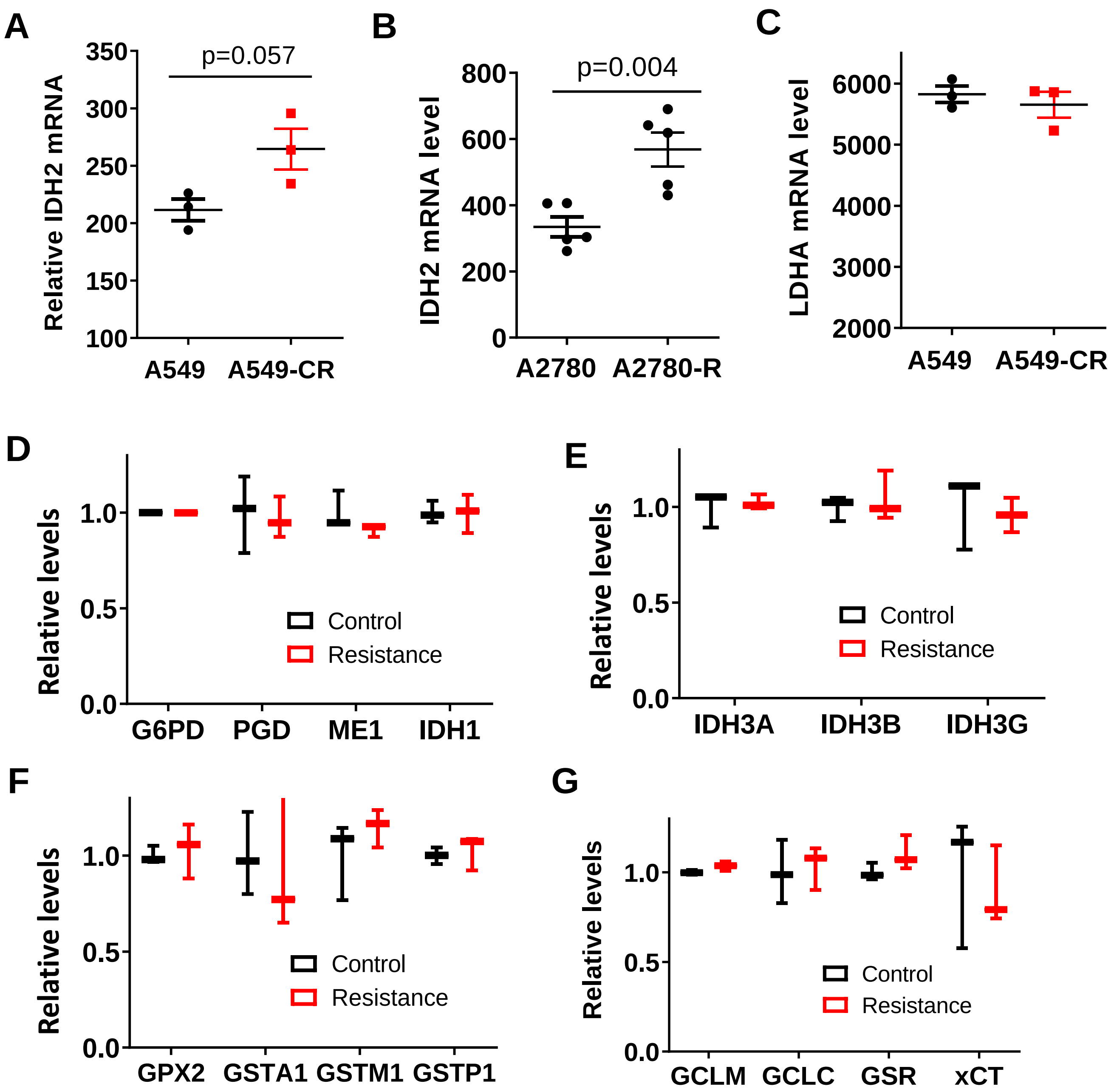

Figure S2. Gene expression in cisplatin-resistant cells in comparison with parental cells. (A) IDH2 mRNA expression in A549 cells and cisplatin-resistant A549-CR cells was compared using GEO dataset (GDS3101); (B) IDH2 mRNA expression in A2780 cells and drug-resistant A2780-R cells was compared using GEO dataset (GDS31754);(C) LDHA mRNA expression in A549 cells and cisplatin-resistant A549-CR cells was compared using GEO dataset (GDS3101); (D-G) Relative mRNA levels of the indicated genes in A549 parental and cisplatin-resistant cells were compared, using the datasets (GDS3101, GDS5247) from GEO database.

Figure S3

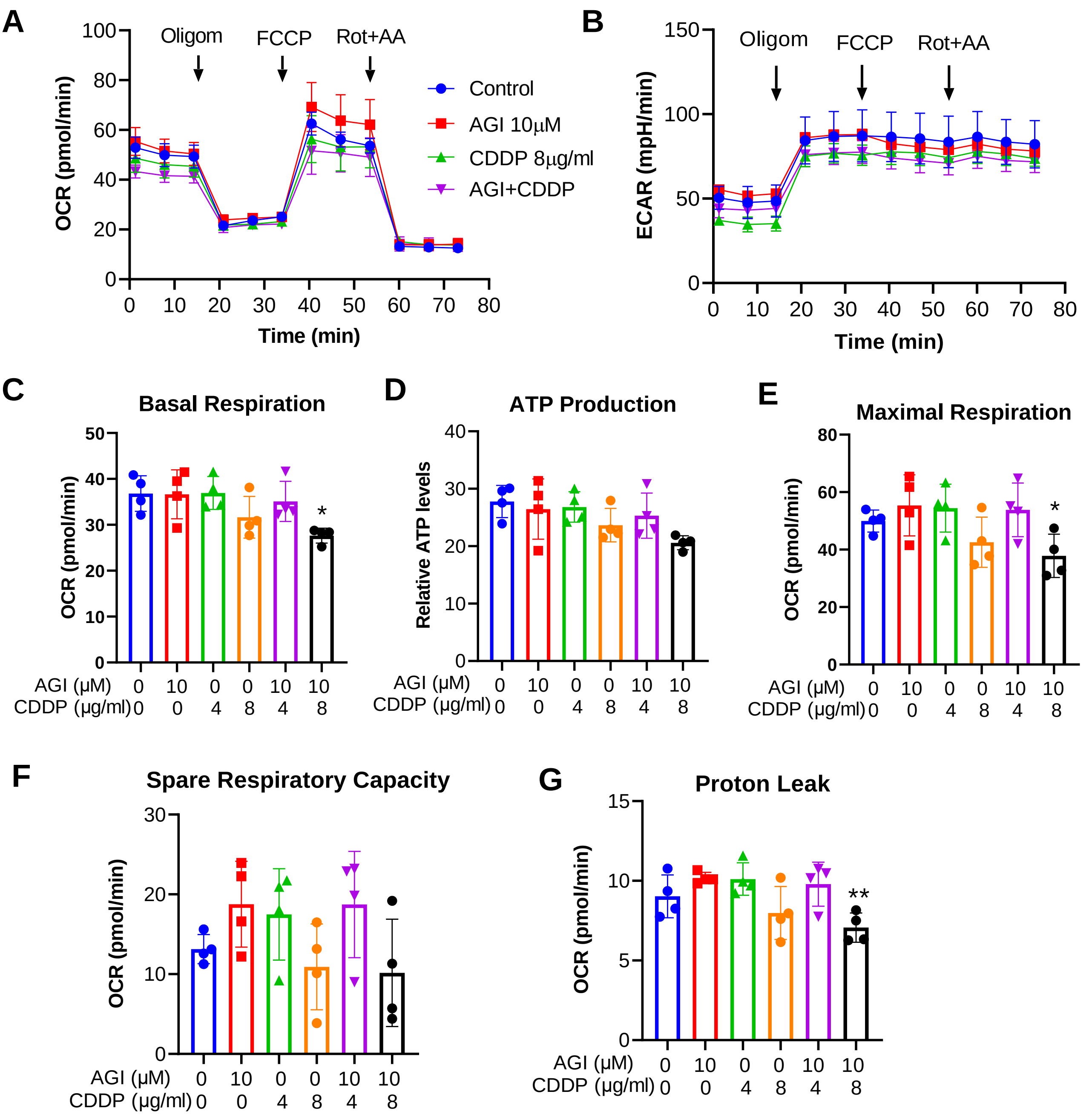

Figure S3. Effect of the IDH2 inhibitor AGI-6780 and cisplatin on cellular metabolism. (A, B) Measurement of cellular oxygen consumption rate (OCR) and extracellular acidification rate (ECAR) in A549 cells treated with AGI6780 (10 μM), CDDP (8μg/ml), or their combination, using a Seahorse XF24 metabolic analyzer. (C) Basal respiration of A549 cells treated with the indicated concentrations of cisplatin and AGI-6780. (D) Mitochondrial ATP production in A549 cells treated with the indicated concentrations of cisplatin and AGI-6780. (E) Maximal respiration capacity in A549 cells treated with the indicated concentrations of cisplatin and AGI-6780. (F) Spared respiratory capacity of A549 cells treated with the indicated concentrations of cisplatin and AGI-6780. (G) Proton leak in A549 cells treated with the indicated concentrations of cisplatin and AGI-6780.

Figure S4

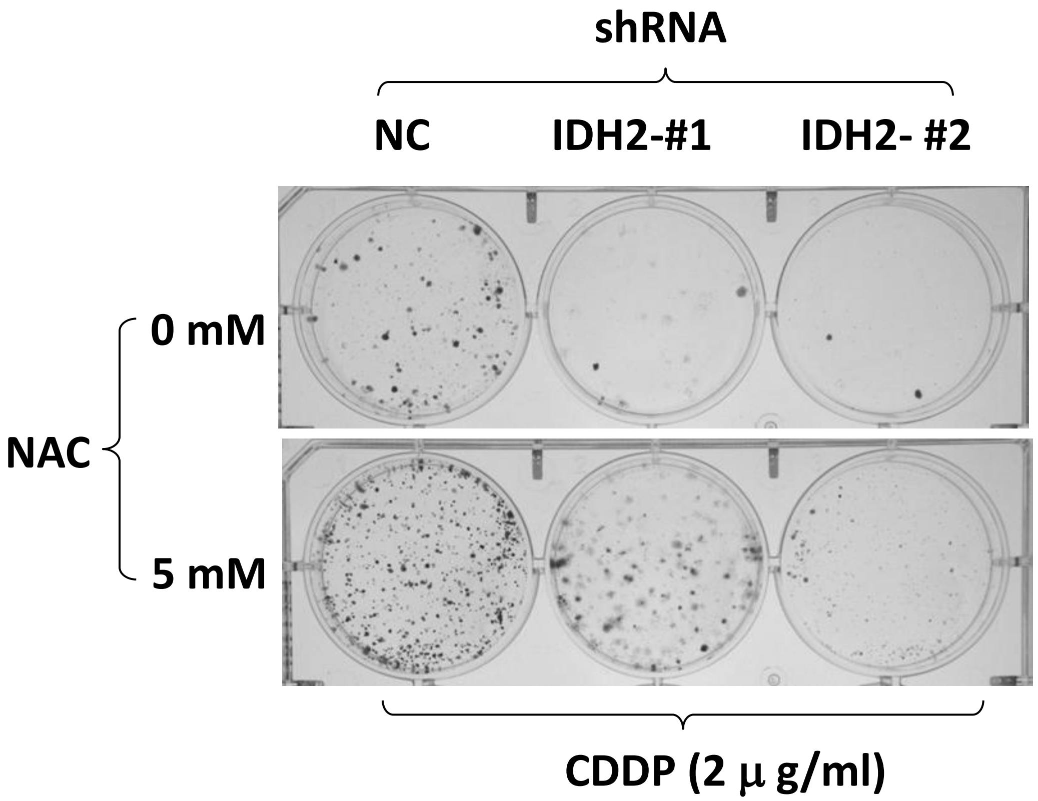

Figure S4. Effect of N-Acetyl-l-Cysteine (NAC) on the cytotoxic effect of cisplatin in lung cancer cells with or without IDH2 knockdown. A549 cells stably transfected with IDH2 shRNA (#1 & #2) or with control shRNA (NC) were pre-treated with the indicated concentration of NAC for 1 hour, and then incubated with 2 μg/ml cisplatin. Cell colonies were fixed and stained on day 14.
